# Prospective cohort study of patients with advanced cancer and their relatives on the experienced quality of care and life (eQuiPe study): a study protocol

**DOI:** 10.1186/s12904-020-00642-w

**Published:** 2020-09-09

**Authors:** Janneke van Roij, Myrte Zijlstra, Laurien Ham, Linda Brom, Heidi Fransen, Art Vreugdenhil, Natasja Raijmakers, Lonneke van de Poll-Franse, eQuiPe study group, Art Vreugdenhil, Maggy Youssef-ELSoud, Geert-Jan Creemers, Ben van den Borne, Wouter de Jong, Arnold Baars, Marieke van den Beuken - van Everdingen, Evelien Kuip, René Bunnik, Mathijs Hendriks, Caroline Mandigers, Jean-Paul van Basten, Vivian van Kampen – van den Boogaart, Philo Werner, Lia van Zuylen, Alexander de Graeff, Anne van Lindert, Marcel Soesan, Jarmo Hunting, Arno Smals, Linda van de Winkel, Gerben Stege, Liese Verhaert, Natascha Peters, Manon Pepels, Tineke Smilde, Peter Nieboer, Sander de Hosson, Marien den Boer, Cordula Pitz, Rick Heyne, Manuel Tjin-A-Ton, Annemieke van der Padt – Pruijsten, Paul van den Berg, Frans Krouwels, Lobke van Leeuwen-Snoeks, Femke van der Meer, Allert Vos, Gerrit Jan Veldhuis, Boelo Poppema, Martine Thijs-Visser, Roxane Heller-Baan, Marjolein van Laren, Karen Maassen van den Brink, Gea Douma, Jeroen Kloover, Dirkje Sommeijer, Lemke Pronk, Ellen Janssens - van Vliet, Lilly-Ann van der Velden, Emma Hafkamp, Henk Codrington, Svitlana Tarasevych, Aart van Bochove, Annemieke van der Padt – Pruijsten, Jaap de Boer, Geraldine Vink

**Affiliations:** 1 The Netherlands Comprehensive Cancer Organization, PO Box 19079, 3501 DB Utrecht, The Netherlands; 2 CoRPS - Center of Research on Psychology in Somatic Diseases, Department of Medical and Clinical Psychology, Tilburg University, Tilburg, The Netherlands; 3 Netherlands Association for Palliative Care (PZNL), Utrecht, the Netherlands; 4 Department of Psychology, Pantein, Boxmeer, The Netherlands; 5 Department of Internal Medicine, St. Jans Gasthuis, Weert, The Netherlands; 6 Department of Medical Oncology, Maxima Medical Centre, Eindhoven, The Netherlands; 7 Division of Psychosocial Research and Epidemiology, The Netherlands Cancer Institute, Amsterdam, The Netherlands

**Keywords:** Study protocol, Prospective study, Longitudinal cohort study, Quality of care, Quality of life, Palliative care, Advanced cancer, Relatives

## Abstract

**Background:**

Palliative care is becoming increasingly important because the number of patients with an incurable disease is growing and their survival is improving. Previous research tells us that early palliative care has the potential to improve quality of life (QoL) in patients with advanced cancer and their relatives. According to limited research on palliative care in the Netherlands, patients with advanced cancer and their relatives find current palliative care suboptimal. The aim of the eQuiPe study is to understand the experienced quality of care (QoC) and QoL of patients with advanced cancer and their relatives to further improve palliative care.

**Methods:**

A prospective longitudinal observational cohort study is conducted among patients with advanced cancer and their relatives. Patients and relatives receive a questionnaire every 3 months regarding experienced QoC and QoL during the palliative trajectory. Bereaved relatives receive a final questionnaire 3 to 6 months after the patients’ death. Data from questionnaires are linked with detailed clinical data from the Netherlands Cancer Registry (NCR). By means of descriptive statistics we will examine the experienced QoC and QoL in our study population. Differences between subgroups and changes over time will be assessed while adjusting for confounding factors.

**Discussion:**

This study will be the first to prospectively and longitudinally explore experienced QoC and QoL in patients with advanced cancer and their relatives simultaneously. This study will provide us with population-based information in patients with advanced cancer and their relatives including changes over time. Results from the study will inform us on how to further improve palliative care.

**Trial registration:**

Trial NL6408 (NTR6584). Registered in Netherlands Trial Register on June 30, 2017.

## Background

Death comes to us all. In 2017, almost 47.000 people died of cancer in the Netherlands, which was with 31% the most common cause of death, followed by cardiovascular disease (25%) and mental disorders or diseases of the nervous system (14%) [1]. The number of people who die of cancer is relatively stable over time [2], despite increasing incidence of cancer and new treatment modalities in cancer, such as immunotherapies and targeted therapies. Fortunately, early detection and advances in cancer treatments have greatly improved survival. Consequently, the time patients live after their diagnosis of advanced cancer is prolonged and the number of patients diagnosed with advanced cancer has increased.

The disease trajectory of advanced cancer for patients is often depicted as a chronic illness, eventually followed by a steep decline and an inevitable death [3]. For relatives of patients with advanced cancer, the disease trajectory also includes a bereavement period after the death of a loved one. At some point in the advanced cancer trajectory, palliative care becomes important. Palliative care is an approach that provides prevention and relief of suffering by means of early identification and assessment and treatment of pain and other physical, psychosocial and spiritual problems [4]. Ideally, palliative care is timely and gradually integrated in oncological care so patients and relatives benefit most from palliative care services [3]. It is important that palliative care is timely integrated in standard oncological care because quality of life (QoL) is improved when patients with advanced cancer receive early palliative care [5–8].

Despite rapid developments [9], the integration and quality of palliative care in oncological care in the Netherlands could be further improved. Recent research shows that patients with advanced cancer are only reasonably satisfied with hospital care [10, 11]. This is worrisome, as the study by Engel et al. suggests that the experienced quality of care (QoC) and QoL may be positively associated. The effect evaluation of the Dutch National Quality Improvement Program Palliative Care showed that most patients and relatives are satisfied with palliative care, but improvements regarding psychosocial and spiritual support and post-bereavement care for relatives are needed [12]. Other research among relatives of patients who died in a University hospital showed that bereaved relatives reported a broad range of experiences, which suggest a widespread variance of the QoC [13]. For instance, Witkamp et al. showed that only 64% of bereaved relatives reported that they had been told that the patient’s death was imminent and 53% stated that the patients’ symptoms and problems in the last 24 h had been sufficiently alleviated. The same study found that according to bereaved relatives, only 42% of the patients had been sufficiently involved in medical decision making [13]. Unfortunately, solid and conclusive information on the experienced QoC and QoL in patients with advanced cancer and their relatives is scarce. Moreover, longitudinal research during the advanced cancer trajectory in patients and relatives is lacking.

A prospective longitudinal observational cohort study on experienced QoC and QoL in patients with advanced cancer and their relatives in the Netherlands is needed. This study will provide more insight into the care experiences, needs and QoL of patients with advanced cancer and their relatives that can guide us in improving daily oncological care and the integration of palliative care.

## Methods

### Aim

The aim of this study is to gain insight into the care experiences and QoL of patients with advanced cancer and their relatives. The following research questions will be addressed:

- What is the experienced QoC according to patients with advanced cancer and their relatives?
- What is the experienced QoL in patients with advanced cancer and their relatives?
- Which factors are associated with the experienced QoC and QoL in patients with advanced cancer and their relatives?

### Study design

The study is a prospective longitudinal observational cohort study on experienced QoC and QoL in patients with advanced cancer and their relatives (eQuiPe study). Patients and their relatives are invited to complete questionnaires on experienced QoC and QoL every 3 months until death. Three to 6 months after a patient is deceased, the bereaved relative will receive a short final questionnaire. The survey data will be directly linked to the detailed clinical data routinely collected on patient characteristics, tumour characteristics, and treatment from the Netherlands Cancer Registry (NCR).

### Setting

The eQuiPe study is a nationwide study that is conducted in multiple hospitals (*n* = 40) in the Netherlands. Per hospital, the departments of medical oncology, pulmonology, and/or urology are participating in the study to identify eligible patients between November 2017 and January 2020.

### Study population

All patients with a diagnosis of a solid metastasized tumor (stage IV) are eligible for inclusion. Additional inclusion criteria are required for patients diagnosed with breast cancer and with prostate cancer to reduce variation and overrepresentation of patients with advanced cancer with a relatively good prognosis. Patients diagnosed with breast cancer are eligible when their metastases are located in multiple organ systems. Patients suffering from prostate cancer are eligible when their cancer is metastasized and castrate-resistant. These criteria are based on information regarding the mean survival time of these groups (NCR). Relatives of included patients, as chosen by the patient, will also be invited to participate in the study. Patients or relatives can participate in the study irrespective of the participation of the other. Patients are also allowed to invite more than one relative to participate in the study. Table 1 provides an overview of the inclusion and exclusion criteria.

**Table 1:** Inclusion- and exclusion criteria

**Inclusion criteria:**	
Patients are eligible for inclusion if they are;	• diagnosed with (progression of) a solid tumour (stage IV) with metastases • additional criteria are in place for the following diagnosis: - breast cancer (stage IV with metastases in multiple organ systems) - prostate cancer (stage IV and Castrate-Resistant) • older than 18 years • able to complete a Dutch self-report questionnaire • able to understand the objective of the study and have signed the informed consent
Relatives of patients are eligible for inclusion if they are;	• indicated by the patient as relative • older than 18 years • able to complete a Dutch self-report questionnaire • able to understand the objective of the study and have signed the informed consent
**Exclusion criteria:**	
Patients and their relatives are excluded for participation in the study if;	• they suffer from dementia • they have a history of severe psychiatric illness

### Recruitment

Health care professionals of participating hospitals will identify patients who meet the inclusion and exclusion criteria. Health care professionals will hand out a patient information leaflet and ask eligible patients if they may be approached by the research team. The patient information leaflet will include comprehensive and understandable information regarding the study. Health care professionals will hand over the patients’ name and phone number to the research team after receiving consent from the patient, which is noted in the patient file or noted on a research sheet. These contact details will be given by phone, secured email or an online secured shared document, whichever route is preferred by the hospital. There is a possibility of self-referral for patients with advanced cancer and their relatives. Advertisement is spread via a Dutch online platform for patients and relatives who are confronted with cancer (www.kanker.nl). Patients and relatives can leave a contact request for the researcher. The recruitment procedure is similar for patients referred by their health care professional.

### Study procedures

#### Inclusion

**Fig. 1:**
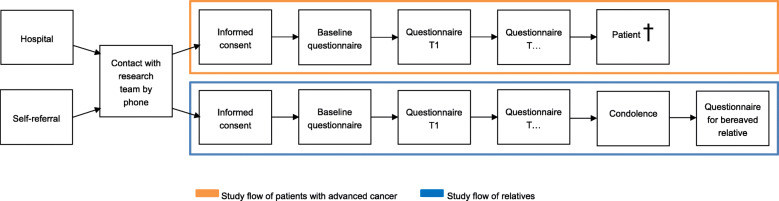
Flowchart of study process

A flowchart of the study procedures are presented in Fig. 1. Within a few days after receiving the patient’s contact information a researcher will phone the patient to explain the study and discuss participation. The patient is asked whether the researcher may approach one of his/her relatives. The relative is informed about the study via a similar procedure. When the patient and/or relative are willing to participate in the study, they are given the option to choose for the informed consent and questionnaires on paper or a web-based version via the Patient Reported Outcomes Following Initial treatment and Long-term Evaluation of Survivorship (PROFILES) registry [14].

#### Data collection

For the web-based version: after giving consent, participants will receive a letter that contains a link to a secure website (www.profielstudie.nl) where the patient can complete the web survey with their own login codes. The login codes are not directly linked to the patient. Patients who complete the online questionnaire can stop and save their data at each desired moment so they can continue the questionnaire at another time. If the participant prefers a paper version of the survey, they receive a paper version with a stamped self-addressed envelope to return the questionnaire to the researcher. If patients do not complete their questionnaire within 2 weeks, a reminder via email or letter will be sent, including the questionnaire. If the patient does not complete their questionnaire within 2 weeks after the reminder, they will be contacted by phone. A questionnaire regarding QoC and QoL will be sent in the same manner every 3 months, till participants indicate that they no longer want to participate in the study, or until death. After the death of a patient, the participating relative will receive our condolences by a personal postcard. Three to 6 months after the death of the patient, the participating bereaved relative will receive a final request to complete the last questionnaire regarding his or her experiences with care in the last phase of life of their loved one, QoL, the patient’s quality of death, and post-bereavement needs and support.

#### Questionnaires

A qualitative study was conducted (METC16.2050) to gather input from patients with advanced cancer and their relatives in the development of our questionnaire [15] (Van Roij et al: Shared persepctives of patients with advanced cancer and their informal caregivers on essential aspects of health care: a qualitative study, submitted). Participants of the focus groups and interviews shared their experiences regarding QoC and QoL, which helped us identify relevant themes for this cohort study. Therefore, the questionnaires involve many topics related to QoC and QoL that were raised by patients with advanced cancer and relatives themselves. Additionally, a systematic literature review was conducted to identify suitable and valid measurement instruments to use in our study [16]. Furthermore, the approved Dutch Quality framework regarding palliative care [17] has also been taken into account while selecting relevant measurement instruments for the eQuiPe study to maximize the comparability of our results. Subsequently, socio-demographic variables such as marital status, ethnicity, educational level, and religion were self-administered and added to the questionnaires. Table 2 provides an overview of all measurement instruments included in the study.

**Table 2:** Overview measurement instruments and times points at which the questionnaires are administered during the study

Measurement	Measurement instrument	Baseline	Follow-up (every 3 months)	After patients’ death
***Patients***				
**Quality of care**	QLQ-IN-PATSAT32 [18], items CQ-index [19], items based on Dutch Quality framework Palliative Care [17]	X	X	
Health care consumption	Self-administered items	X	X	
Shared decision making	CPS [20], DEPS [21], Self-administered items	X	X	
Health care needs	PNPC-sv patient form [22]	X	X	
**Quality of life**	EORTC QLQ-C30 [23]	X	X	
Spiritual wellbeing	FACIT-sp [24]	X	X	
Social support	FACT-G scale [25]	X	X	
Use of social network	Self-administered item	X	–	
Sexual health	single items EORTC	X	X	
Body image	BIS [26]	X	X	
Relationship satisfaction^a^	Relationship ladder of the DAS [27]	X	X	
Illness perception	BIP [28]	X	X	
Individual coping	Brief COPE Inventory [29]	X	X	
Resilience	Connor-Davidson Resilience Scale [30]	X	X	
Dyadic coping^a^	DCI [31]	X	–	
Self-management	HeiQ [32]	X	–	
Depression	HADS depression scale [33]	X	–	
***Relatives***				
**Quality of care**	INPATSAT32 [18], CQ-index [19], items based on Dutch Quality framework Palliative Care [17]	X	X	–
Health care consumption	Self-administered items	X	X	
Health care needs	PNPC-sv caregiver form [22]	X	X	–
Evaluation of services	VOICES-SF [34], items based on Dutch Quality framework Palliative Care [17]	–	–	X
**Quality of life**	EORTC QLQ-C30 items [23]	X	X	X
Sexual health	single items EORTC	X	X	–
Social support	FACT-G scale [25]	X	X	X
Personal self-care	Personal Self-Care subscale of the SCPS [35]	X	X	X
Caregiver burden	ZARIT-12 [36], SRB [37]	X	X	–
Relationship satisfaction^a^	Relationship ladder from the DAS [27]	X	X	–
Individual coping	brief COPE Inventory [29]	X	X	–
Resilience	Connor-Davidson Resilience Scale [30]	X	X	–
Pre-death grief	Pre-death grief [38]	X	X	–
Dyadic coping^a^	DCI [31]	X	–	–
Circumstances of death	Self-administered items	–	–	X
Openness of communication about illness and death	CCID [39]	–	–	X
Impact of death	IES [40]	–	–	X

*Abbreviations*: *BIP* Brief illness perception, *BIS* Body image scale, *BMI* Body mass index, *CCID* Caregiver’s communication with the patient about illness and death, *CPS* Control preferences scale, *CQ-index* Consumer quality index, *DAS* Dyadic adjustment scale, *DCI* Dyadic coping inventory, *DEPS* Decision-making participation self-efficacy scale, *EORTC QLQ-C30* European organization for research and treatment of cancer quality of life questionnaire core 30 items, *FACIT-sp* Functional assessment of chronic illness therapy spiritual well-being, *FACT-G* Functional assessment of cancer therapy general, *HADS* Hospital anxiety and depression scale, *HeiQ* Health education impact questionnaire, *IES* Impact of event scale, *INPATSAT32* In-patient satisfaction with care measure 32 items, *PNPC-sv* Problems and needs in palliative care short form, *SCPS* Self care practices scale, *SRB* Self-rated burden scale, *VOICES-SF* Views of informal carers’ evaluation of services short form, *ZARIT-12* Zarit Burden

^a^ only provided to those patients and relatives with a partner

The questionnaires were tested on completion time, appropriateness, and burden in a pilot study (*n* = 31) among patients with advanced cancer and relatives. The pilot consisted of the ‘think-aloud’ method with six participants (two patients with advanced cancer, two relatives, and two bereaved relatives) and 15 participants gave postal feedback. Results of the pilot study indicated that the mean completion time for the most extensive questionnaire (baseline measurement for patients) was 38 min and completing the questionnaire was not experienced as a great burden, confrontational, incomprehensible, or inappropriate. Suggestions made by the participants of the pilot study were taken into account to further improve the questionnaire. Results from the pilot suggested that the questionnaire length is suitable for our study population. For patients who also participate in national tumor-specific cohort studies (PLCRC, POCOP, PACAP) [41], the questionnaires will be aligned and adjusted in order to decrease the response burden for participants.

#### PROFILES and NCR

PROFILES will be used for the logistics of the questionnaires. PROFILES is a registry for the study of the physical and psychosocial impact of cancer and its treatment from a dynamic, growing population-based cohort of people confronted with cancer. PROFILES follows the quality guidelines that are formulated in the ‘Data Seal of Approval’ document (www.datasealofapproval.org), developed by Data Archiving and Networked Services. The PROFILES registry is an ongoing data collection of patient reported outcomes within the sampling frame of the NCR and can be linked with clinical data of all individuals newly diagnosed with cancer in the Netherlands. For the eQuiPe study, socio-demographic and clinical data will be obtained from the NCR. Socio-demographic variables include date of birth, sex, and socio-economic status. Clinical data include cancer type, stage, and date of diagnosis.

### Study parameters

#### Main outcome

The main outcome of this study is the experienced QoC and QoL in patients with advanced cancer and their relatives. This includes all domains of QoL such as physical, psychological, social, and spiritual wellbeing.

#### Secondary outcomes

Secondary outcomes in this study are health care needs, shared decision making, and health care consumption of patients and relatives. Furthermore, social support, resilience, body image, sexual wellbeing, illness perception, individual coping, self-management, depression and use of social networks are measured (Table 2). Relatives will also receive questions on caregiver burden and personal self-care. For patients and relatives with a partner, also relationship satisfaction and dyadic coping will be assessed. In bereaved relatives, health care services in the last days of the patients’ life and aftercare will be evaluated. Furthermore, circumstances and impact of the patient’s death and the communication between relative and patient about illness and death are assessed.

### Statistical analysis

We aim for a large study population of approximately 1500 patients with advanced cancer and 1000 relatives. Including a large group of patients and relatives is necessary to assess the QoC and QoL of these participants at different time points in the palliative care trajectory and its course. A study sample of this size enables us to perform subgroup analyses, for example per age group, primary tumor site, cancer treatment, diagnosis, sex, and geographical region. Also, high dropout and lower response rates due to disease-related characteristics of our study population have to be taken into account. Due to the nature of this observational study, no sample size calculations haven been performed but the number of patients are based on annual incidence of advanced cancer in the Netherlands as recorded in the NCR.

All statistical analysis will be performed using statistical packages STATA version 16. For all analyses a two-sided significance level of *p* < 0.05 will be used. Descriptive statistics (frequencies, median, mean) will be used to analyze the experienced QoC, QoL, healthcare use, advance care planning, symptom burden of patients and relatives. Further, univariate analyses will be used to analyze the crude differences between subgroups regarding QoC or QoL using parametric tests, provided that the assumptions of these tests are met. If not, non-parametric tests will be used. When testing differences between subgroups, we will adjust for confounders which are theoretically relevant and statistically associated with the outcome variable of interest. Additionally, multi-level analyses will be used to analyze the primary and secondary outcomes over time.

### Dissemination

The funding party (Roparun) and accredited METC of this study will receive a final report of the study with recommendations. Furthermore, results of this study will be published in multiple peer-reviewed publications in scientific journals. The study aims to provide an (inter-)nationally accessible source of data. These data will be available for (internal) auditing and policy making, as all data of the PROFILES registry. PROFILES will perform first analyses on the data to check the quality and validity. After this process, the data will be freely available for research questions from other non-commercial groups in the Netherlands and abroad, subject to study question, privacy, and confidentiality restrictions, and registration [14].

## Discussion

The eQuiPe study aims to gain more insight into the experienced QoC and QoL in patients with advanced cancer and their relatives. Results from the study will raise awareness regarding the poor prognosis of advanced cancer and palliative care needs of patients and their relatives. Furthermore, the eQuiPe study is a unique national project in which many health care professionals unite to gain a deeper understanding of experienced palliative care. Results from this study will inform us on how to further improve palliative care in the Netherlands for patients with advanced cancer and their relatives.

This prospective longitudinal observational cohort study has several strengths. First, we will include about 1500 patients with solid metastasized tumor of any type and approximately 1000 relatives. Due to this large study population it is possible to assess the experienced QoC and QoL of patients and relatives at different time points in the palliative care trajectory. Moreover, this large study sample also enables us to perform subgroup analyses, for example per age group, primary tumor site, cancer treatment, diagnosis, sex, and geographical region. Second, both advanced cancer patients and their relatives are included. Our explorative qualitative study on QoL in patients with advanced cancer and their relatives, as preparation for this current study, showed that advanced cancer has a substantial impact on social engagement, social identity, and social networks for both patients and relatives [15]. Therefore, in order to improve palliative care it is of essence to focus on relatives to really comprehend their experiences. The inclusion of patients and relatives simultaneously also gives rise to the opportunity to assess them as a dyad, thus taking the interaction between patients and relatives into account. Third, the eQuiPe study is a longitudinal study. In contrast to the majority of the conducted studies on palliative care, patients and relatives will now be followed over time, from inclusion until death and thereafter for the relatives. This will provide insight in changes in their experiences over time which are currently only limitedly known to us. Fourth, our approach of including patients and relatives is highly personal. All patients and relatives will be contacted by phone by the research team to discuss participation. Participants will also be contacted by phone when they have not completed one of the questionnaires. At last, this is a national study. Already 40 of the 80 hospitals in the Netherlands are collaborating with the eQuiPe study, covering a range of academic, teaching and general hospitals and the study has a good geographic spread. Therefore, the conclusions that will follow from the results of the eQuiPe study are likely to be representative for the Netherlands and generalizable for different regions and care settings.

We also expect to encounter some challenges and potential limitations in the eQuiPe study. Firstly, selection bias cannot be ruled out because patients with a higher QoL may be more likely to participate in the study compared to patients with a lower QoL [42]. Health care professionals may contribute to this bias by only asking patients with a higher QoL to participate in our study but also patients that are self-referred may be more inclined to participate when having a higher QoL. For this reason, we emphasize during the initiation visit that professionals can ask *all patients* with metastasized disease who fulfill the inclusion criteria. Furthermore, attrition may occur because the condition of the patient might worsen over time such that further participation becomes impossible. As a result, information on the last months of life may be limited. Besides that, the life expectancy of patients varies depending on primary tumor type, which means that some patients will live for 3 months while others may live much longer. In an attempt to reduce this variation and overrepresentation of patients with advanced cancer with a relatively good prognosis, additional inclusion criteria are required for patients with breast cancer and with prostate cancer. A possible alternative for the starting point we considered was the surprise question: “Would I be surprised if this patient died in the next 12 months?”. However, according to the review of Downar at al [43]., the surprise question seems to be a poor to modestly predictive tool for patients with a near death. Therefore, we opted for an objective measure, namely having metastatic cancer. Another possible limitation is the length of the questionnaires. Due to the length, the workload for participants can become high, which can lead to a higher drop-out, especially in patients experiencing more symptoms from their disease. However, a meta-analysis showed no clear indication that response rates are attributable to the length of questionnaires [44]. A possible solution could be to use computer adaptive testing, but when using computer adaptive testing, it is of essence that all participants use the same mode (i.e., a computer) to answer the questionnaires, otherwise scores are not comparable. We wanted participants to have the option to complete questionnaires on paper as this remains a commonly preferred mode of participation [45]. At last, the clinical data are collected by the NCR, but these are mostly based on initial diagnosis and treatment. Therefore, some clinical data, for example information about treatment in the complete palliative care trajectory, will be collected via the questionnaires. However, some patients may not be fully aware of the specifics of the treatment they receive, hence, information regarding these clinical data may be incomplete.

## Data Availability

Data sharing is not applicable to this article as no new data were created or analyzed in this study.

## Abbreviations

QoC: Quality of care
QoL: Quality of life
NCR: National Cancer Registry
PI: Principle investigator
PROFILES: Patient Reported Outcomes Following Initial treatment and Long term Evaluation of Survivorship
